# 

**DOI:** 10.1192/bjb.2024.10

**Published:** 2024-08

**Authors:** Shivani Patel

**Affiliations:** East London NHS Foundation Trust, London, UK. Email: shivaninpatel183@gmail.com

**Figure:**
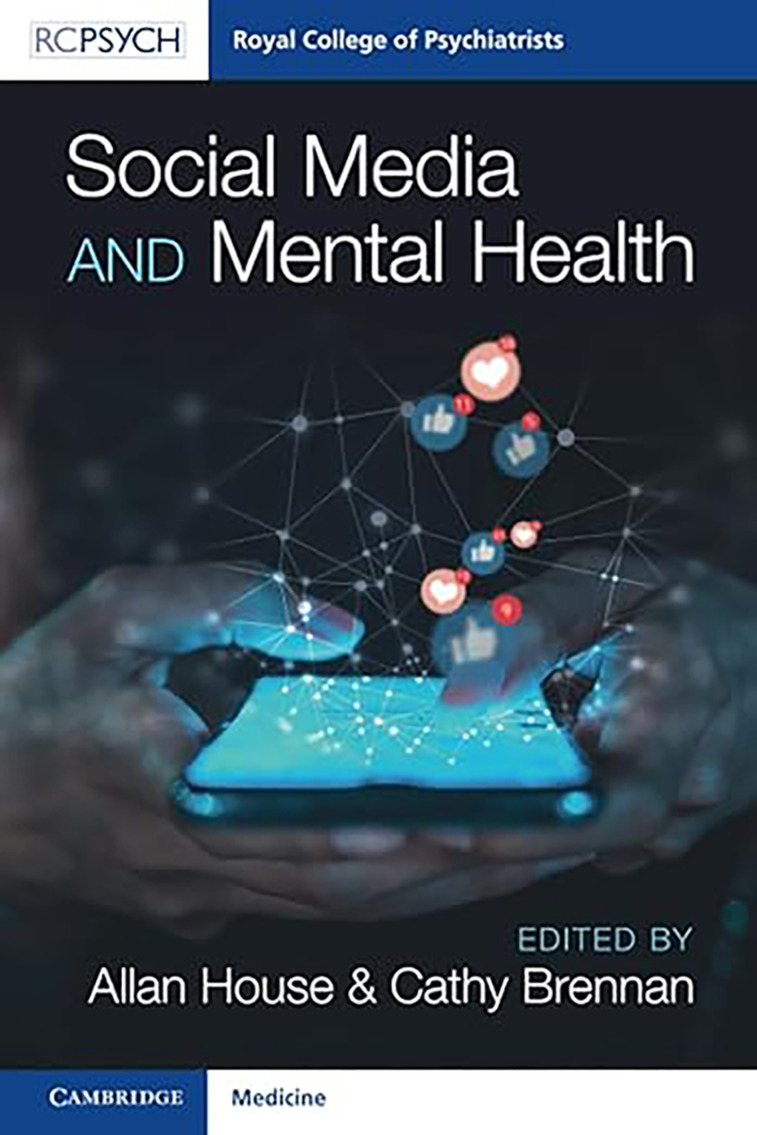

The relationship between social media and mental health has been a highly contested area over the past decade, and has indeed generated significant debate among clinicians and politicians alike. Issues regarding the use of social media among young people have been of particular concern, and over the past few years the Royal College of Psychiatry has called for psychiatrists to routinely ask about the use of technology within assessments, and to consider the risks that social media may pose to mental health. As such, this book provides a timely summary of our current understanding of social media and how use of these platforms may have clinical implications.

The book is divided into three sections. The first section, entitled ‘Understanding Social Media’, provides an overview as to how social media platforms were initially developed and have since evolved. The authors additionally discuss the current legal and ethical frameworks that relate to social media use in the UK, and areas where this may need to develop further. The second section of the book is entitled ‘Social Media and Mental Health’. This section is of particular clinical interest as it explores the impact of social media on a range of presentations including mood disorders, eating disorders and gambling. The authors provide a well considered and balanced discussion of the evidence relating to the impact of social media in these clinical areas. In the final section, ‘Social Media as a Resource’, the authors deliberately choose to end the book on a positive note. This section outlines the progress that has been made in improving online safety and discusses how technology can be leveraged to provide information and interventions for those suffering from mental health conditions.

The development of social media has no doubt had far-reaching implications in several areas of modern life. This book provides an up-to-date and accessible summary of our current understanding of this fast-developing field, and seeks to ensure that clinicians are appropriately prepared to consider how technology may affect the lives of their patients.

